# The Confounding Influence of Older Age in Statistical Models of Telehealth Utilization

**DOI:** 10.5195/ijt.2023.6565

**Published:** 2023-12-12

**Authors:** David Shilane, Heidi Ting'an Lu, Zhenyi Zheng

**Affiliations:** Program in Applied Analytics, School of Professional Studies, Columbia University, New York, New York, USA

**Keywords:** Age, Confounding variables, Stratified models, Telehealth

## Abstract

Older age is a potentially confounding variable in models of telehealth utilization. We compared unified and stratified logistic regression models using data from the 2021 National Health Interview Survey. A total of 27,626 patients were identified, of whom 38.9% had utilized telehealth. Unified and stratified modeling showed a number of important differences in their quantitative estimates, especially for gender, Hispanic ethnicity, heart disease, COPD, food allergies, high cholesterol, weak or failing kidneys, liver conditions, difficulty with self-care, the use of mobility equipment, health problems that limit the ability to work, problems paying bills, and filling a recent prescription. Telehealth utilization odds ratios differ meaningfully between younger and older patients in stratified modeling. Traditional statistical adjustments in logistic regression may not sufficiently account for the confounding influence of older age in models of telehealth utilization. Stratified modeling by age may be more effective in obtainina clinical inferences.

Telehealth is a set of technological tools that facilitate remote health appointments between a patient and a provider (Koonin et al. 2020). The utilization of telehealth has greatly expanded since the beginning of the COVID-19 pandemic (Chang et al. 2021). Telehealth has a great potential to improve the accessibility of health care (Dorsey & Topol, 2016). Barriers to in-person healthcare, which may be based on geography (Francke et al., 2021), language (Dixit et al., 2021), costs (Wood et al., 2021), transportation (Westby et al., 2021), mobility (Forducey et al., 2012), and the required time commitments for an appointment, among others, can all be mitigated with telehealth. Considerable research has been conducted to understand the trends in the recent adoption of telehealth, to identify the barriers to telehealth utilization, and to quantify the disparities in the adoption of telehealth (Smith & Raskin, 2021). Prior literature has established that telehealth utilization varies by age (Chang et al., 2021), gender (Darrat et al., 2021), income (Dixit et al., 2021; Franciosi & Quon, 2021), race (Chunara et al., 2020; Jain et al., 2020; Weber et al., 2020), ethnicity (Chunara et al., 2020; Weber et al., 2020), and geographical region (Jain et al., 2020; Lau et al., 2022; Perzynski et al., 2017). A number of barriers to telehealth, such as conceptual comfort (Odeh, 2015), access to technology (Franciosi & Quon 2021; Sieck 2021), and comfort with technology (Haynes et al. 2021), contribute to disparities in telehealth utilization (Perzynski 2017). As a result, many of the patients who could benefit the most from telehealth are among the least likely to utilize the technology.

Many studies of telehealth adoption are conducted using observational research methods with data gathered from existing health records (Barnett et al., 2018; Mehrotra, 2017; Weber et al., 2020). In this setting, multivariable statistical models are typically applied to estimate associations while adjusting for potentially confounding factors. In many examples of multivariable models of telehealth utilization or its impact on health outcomes, age is incorporated as a controlling variable (Adepoju et al., 2022; Chunara et al., 2020; van der Burg et al., 2020). For example, Adepoju et al. (2022) utilized multivariable modeling to estimate the association between telehealth utilization and factors like insurance coverage and distance to a health clinic while adjusting for the patient's age group and gender. In van der Burg et al. (2020), multivariable models were applied to estimate the associated reduction in hospitalizations for patients with chronic obstructive pulmonary disease (COPD) who utilize telehealth while adjusting for the patient's age and other factors. Similarly, Chunara et al. (2020) applied multilevel regression modeling to estimate the likelihood of telehealth utilization based on demographic and medical factors while adjusting for the patient's age.

The suitability of multivariable statistical models relies upon an assumption of similar trends in different subgroups. For instance, in logistic regression (LR), a confounding variable's coefficient represents the additive increase in the natural logarithm of the odds of success. Then the quality of the model's estimates depends on how accurately the adjustments account for the confounding variables. As an alternative methodology, stratified models can be fit separately in patient subgroups. This allows for differing estimates of the relationships among variables within each subgroup.

Older age is a critical factor in the adoption of telehealth (Chang et al., 2021). Older patients have higher rates of chronic conditions, utilize medical services more frequently, and face greater health risks (Peek, 2016). Older patients are also more likely to have mobility concerns that create challenges for attending in-person clinical appointments. It is well established that older people are less likely to adopt new technologies and exhibit reduced comfort in utilizing technology (Kim et al., 2016). Patients who are at least 65 years old utilize telehealth at greatly reduced rates relative to younger patients (Lau et al. 2022; Weber et al., 2020).

This study investigated the role of older age as a confounding variable in statistical models of telehealth utilization by comparing the modeling strategies of multivariable adjustment for age and stratification based on age. We hypothesize that the likelihood of recent telehealth utilization for older patients, in comparison with younger patients, is influenced by different variables and demonstrates disparities in their quantitative associations. We compared a model that adjusts for older age relative to stratified models. In doing so, we sought to identify variables with quantitative estimates that may be subject to confounding bias based on the modeling strategy.

## Methods

This study relied upon publicly available, de-identified, patient-level data (National Center for Health Statistics 2021) on adults at least 18 years old from the 2021 National Health Interview Survey (NHIS) (National Center for Health Statistics, 2022). The NHIS collected information on a wide range of variables, including demographic factors, health conditions, other health factors, challenges of ability, behavioral health concerns, and social needs. The authors selected variables for statistical analysis based on clinical relevance and completeness of the measured information. Variables with more than 10% missingness were excluded from consideration.

The study's primary outcome was the binary utilization of telehealth (a virtual medical appointment by phone or video) within the past 12 months. The patient's age was categorized into groups (18–34, 35–49, 50–64, 65–74, and at least 75). Other variables, such as race, ethnicity, and the patient's income ratio relative to the poverty line, were consolidated into a smaller number of categories.

We then constructed multivariable logistic regression models of telehealth utilization with the following methods:

- Unified LR: A model including patients of all ages.
- Stratified LR: Under 65: A model including only patients under 65 years old.
- Stratified LR: 65+: A model including only patients at least 65 years old.

For each model, the youngest available age group was utilized as the reference group for the age group variable. Otherwise, these multivariable logistic regression models included the same range of input variables (Tables 1–5). For each logistic regression model, we calculated each variable's estimated odds ratio, 95% confidence interval for the odds ratio, and p-value for a test of the odds ratio relative to a standard of 1. We then qualitatively and quantitatively compared each model's estimates for each variable across the unified and stratified methods. We identified variables that meaningfully and statistically differed in terms of their estimated odds ratios.

**Table 1.** Comparison of the Estimated Odds Ratios for Demographic Variables in the Unified LR, Stratified LR: Under 65, and Stratified LR: 65+ Models

Variable	Unified LR	Stratified LR: Under 65	Stratified LR: 65+	Difference in Odds Ratios
**Age: 18–34**	Reference Group	Reference Group		
**Age: 35–49**	OR: 0.99 (95% CI: 0.91–1.08, p = 0.881)	OR: 0.99 (95% CI: 0.91–1.09, p = 0.906)		
**Age: 50–64**	OR: 0.79 (95% CI: 0.72–0.87, p = 0.000)	OR: 0.79 (95% CI: 0.71–0.87, p = 0.000)		
**Age: 65–74**	OR: 0.73 (95% CI: 0.66–0.81, p = 0.000)		Reference Group	
**Age: 75+**	OR: 0.62 (95% CI: 0.55–0.70, p = 0.000)		OR: 0.87 (95% CI: 0.78–0.96, p = 0.007)	
**Education: No College**	Reference Group	Reference Group	Reference Group	
**Education: Some College**	OR: 1.41 (95% CI: 1.29–1.55, p = 0.000)	OR: 1.43 (95% CI: 1.27–1.60, p = 0.000)	OR: 1.42 (95% CI: 1.22–1.66, p = 0.000)	0
**Education: College Degree**	OR: 1.58 (95% CI: 1.46–1.71, p = 0.000)	OR: 1.61 (95% CI: 1.46–1.78, p = 0.000)	OR: 1.51 (95% CI: 1.33–1.72, p = 0.000)	−0.1
**Education: Graduate Degree**	OR: 1.97 (95% CI: 1.80–2.17, p = 0.000)	OR: 1.99 (95% CI: 1.77–2.24, p = 0.000)	OR: 1.94 (95% CI: 1.66–2.26, p = 0.000)	−0.05
**Male**	OR: 0.80 (95% CI: 0.75–0.84, p = 0.000)	OR: 0.73 (95% CI: 0.68–0.79, p = 0.000)	OR: 0.96 (95% CI: 0.87–1.06, p = 0.433)	0.23
**Race/Ethnicity: White Race, Non-Hispanic Ethnicity**	Reference Group	Reference Group	Reference Group	
**Race/Ethnicity: Black Race**	OR: 1.03 (95% CI: 0.94–1.14, p = 0.493)	OR: 1.03 (95% CI: 0.92–1.15, p = 0.644)	OR: 1.03 (95% CI: 0.86–1.22, p = 0.769)	0
**Race/Ethnicity: Hispanic Ethnicity**	OR: 1.17 (95% CI: 1.07–1.29, p = 0.001)	OR: 1.10 (95% CI: 0.99–1.22, p = 0.074)	OR: 1.59 (95% CI: 1.30–1.95, p = 0.000)	0.49
**Race/Ethnicity: Asian**	OR: 0.94 (95% CI: 0.83–1.06, p = 0.307)	OR: 0.93 (95% CI: 0.81–1.06, p = 0.294)	OR: 0.97 (95% CI: 0.75–1.27, p = 0.843)	0.04
**Race/Ethnicity: AIAN**	OR: 1.18 (95% CI: 0.95–1.47, p = 0.125)	OR: 1.19 (95% CI: 0.92–1.53, p = 0.184)	OR: 1.16 (95% CI: 0.77–1.76, p = 0.474)	−0.02
**Race/Ethnicity: Other/Unknown**	OR: 0.87 (95% CI: 0.70–1.09, p = 0.238)	OR: 0.82 (95% CI: 0.64–1.06, p = 0.131)	OR: 1.06 (95% CI: 0.61–1.83, p = 0.832)	0.24
**Region: Northeast**	Reference Group	Reference Group	Reference Group	
**Region: Midwest**	OR: 0.68 (95% CI: 0.62–0.74, p = 0.000)	OR: 0.68 (95% CI: 0.61–0.76, p = 0.000)	OR: 0.65 (95% CI: 0.56–0.76, p = 0.000)	−0.03
**Region: South**	OR: 0.78 (95% CI: 0.72–0.84, p = 0.000)	OR: 0.74 (95% CI: 0.67–0.82, p = 0.000)	OR: 0.85 (95% CI: 0.74–0.97, p = 0.019)	0.11
**Region: West**	OR: 1.22 (95% CI: 1.12–1.32, p = 0.000)	OR: 1.19 (95% CI: 1.07–1.32, p = 0.001)	OR: 1.27 (95% CI: 1.09–1.47, p = 0.002)	0.08
**Rural Geography**	OR: 0.60 (95% CI: 0.55–0.65, p = 0.000)	OR: 0.58 (95% CI: 0.52–0.64, p = 0.000)	OR: 0.62 (95% CI: 0.54–0.71, p = 0.000)	0.04

*Note*. The values presented here are a subset of the larger model. The Unified LR model was developed on 27,626 adult patients including all ages. The Stratified LR: Under 65 was constructed on 19,340 patients, and the Stratified LR: 65+ was based on 8,286 patients. The data for the model were gathered from the 2021 NHIS study.

**Table 2.** Comparison of the Estimated Odds Ratios for Health Condition Variables in the Unified LR, Stratified LR: Under 65, and Stratified LR: 65+ Models

Variable	Unified LR	Stratified LR: Under 65	Stratified LR: 65+	Difference in Odds Ratios
**Arthritis**	OR: 1.20 (95% CI: 1.12–1.29, p = 0.000)	OR: 1.18 (95% CI: 1.07–1.30, p = 0.001)	OR: 1.26 (95% CI: 1.14–1.39, p = 0.000)	0.08
**Asthma**	OR: 1.20 (95% CI: 1.10–1.29, p = 0.000)	OR: 1.19 (95% CI: 1.09–1.31, p = 0.000)	OR: 1.20 (95% CI: 1.03–1.39, p = 0.019)	0
**CHD**	OR: 1.23 (95% CI: 1.08–1.40, p = 0.002)	OR: 1.54 (95% CI: 1.22–1.96, p = 0.000)	OR: 1.10 (95% CI: 0.94–1.28, p = 0.220)	−0.44
**COPD**	OR: 1.03 (95% CI: 0.91–1.17, p = 0.611)	OR: 0.87 (95% CI: 0.72–1.06, p = 0.162)	OR: 1.18 (95% CI: 1.00–1.38, p = 0.049)	0.3
**Cancer**	OR: 1.30 (95% CI: 1.20–1.42, p = 0.000)	OR: 1.38 (95% CI: 1.21–1.58, p = 0.000)	OR: 1.27 (95% CI: 1.14–1.41, p = 0.000)	−0.11
**Chronic Fatigue Syndrome**	OR: 1.43 (95% CI: 1.12–1.81, p = 0.003)	OR: 1.45 (95% CI: 1.08–1.95, p = 0.015)	OR: 1.36 (95% CI: 0.90–2.04, p = 0.141)	−0.09
**Diabetes**	OR: 1.26 (95% CI: 1.15–1.38, p = 0.000)	OR: 1.33 (95% CI: 1.17–1.52, p = 0.000)	OR: 1.16 (95% CI: 1.02–1.32, p = 0.020)	−0.17
**Dry Mouth**	OR: 1.13 (95% CI: 1.01–1.27, p = 0.038)	OR: 1.14 (95% CI: 0.96–1.35, p = 0.148)	OR: 1.13 (95% CI: 0.97–1.33, p = 0.124)	0
**Food Allergy**	OR: 1.12 (95% CI: 1.03–1.23, p = 0.009)	OR: 1.21 (95% CI: 1.08–1.34, p = 0.000)	OR: 0.92 (95% CI: 0.78–1.09, p = 0.348)	−0.28
**High Cholesterol**	OR: 1.05 (95% CI: 0.99–1.12, p = 0.120)	OR: 1.03 (95% CI: 0.94–1.12, p = 0.549)	OR: 1.11 (95% CI: 1.01–1.23, p = 0.034)	0.09
**Hypertension**	OR: 1.04 (95% CI: 0.97–1.11, p = 0.294)	OR: 1.03 (95% CI: 0.95–1.12, p = 0.462)	OR: 1.08 (95% CI: 0.97–1.20, p = 0.139)	0.05
**Kidneys Weak or Failing**	OR: 1.46 (95% CI: 1.25–1.70, p = 0.000)	OR: 1.26 (95% CI: 0.98–1.60, p = 0.067)	OR: 1.58 (95% CI: 1.30–1.91, p = 0.000)	0.32
**Liver Condition**	OR: 1.22 (95% CI: 0.93–1.60, p = 0.161)	OR: 1.36 (95% CI: 0.96–1.91, p = 0.079)	OR: 1.05 (95% CI: 0.66–1.67, p = 0.835)	−0.31
**MI/Heart Attack**	OR: 1.03 (95% CI: 0.88–1.21, p = 0.702)	OR: 0.93 (95% CI: 0.70–1.22, p = 0.588)	OR: 1.06 (95% CI: 0.87–1.29, p = 0.562)	0.13
**Stroke**	OR: 0.98 (95% CI: 0.84–1.14, p = 0.813)	OR: 0.88 (95% CI: 0.68–1.15, p = 0.360)	OR: 1.04 (95% CI: 0.87–1.25, p = 0.658)	0.16

*Note*. The values presented here are a subset of the larger model. The Unified LR model was developed on 27,626 adult patients including all ages. The Stratified LR: Under 65 was constructed on 19,340 patients, and the Stratified LR: 65+ was based on 8,286 patients. The data for the model were gathered from the 2021 NHIS study.

**Table 3.** Comparison of the Estimated Odds Ratios for Other Health Variables in the Unified LR, Stratified LR: Under 65, and Stratified LR: 65+ Models

Variable	Unified LR	Stratified LR: Under 65	Stratified LR: 65+	Difference in Odds Ratios
**BMI: Normal Weight or Underweight**	Reference Group	Reference Group	Reference Group	
**BMI: Overweight**	OR: 1.01 (95% CI: 0.94–1.08, p = 0.733)	OR: 1.02 (95% CI: 0.94–1.11, p = 0.570)	OR: 1.00 (95% CI: 0.90–1.13, p = 0.952)	−0.02
**BMI: Obese**	OR: 1.06 (95% CI: 0.99–1.14, p = 0.105)	OR: 1.06 (95% CI: 0.97–1.15, p = 0.208)	OR: 1.10 (95% CI: 0.97–1.25, p = 0.146)	0.04
**BMI: Unknown**	OR: 1.00 (95% CI: 0.82–1.21, p = 1.000)	OR: 1.06 (95% CI: 0.83–1.34, p = 0.658)	OR: 0.90 (95% CI: 0.65–1.26, p = 0.542)	−0.15
**Hospitalized in the Past 3 Months**	OR: 1.44 (95% CI: 1.30–1.60, p = 0.000)	OR: 1.54 (95% CI: 1.35–1.77, p = 0.000)	OR: 1.32 (95% CI: 1.13–1.54, p = 0.001)	−0.23
**No Usual Facility for Medical Care**	OR: 0.47 (95% CI: 0.41–0.53, p = 0.000)	OR: 0.47 (95% CI: 0.41–0.53, p = 0.000)	OR: 0.57 (95% CI: 0.39–0.82, p = 0.003)	0.1
**Number of ED Visits in the Past 12 Months**	OR: 1.09 (95% CI: 1.04–1.14, p = 0.000)	OR: 1.07 (95% CI: 1.01–1.13, p = 0.012)	OR: 1.14 (95% CI: 1.05–1.23, p = 0.001)	0.06
**Number of Urgent Care Visits in the Past 12 Months**	OR: 1.09 (95% CI: 1.06–1.12, p = 0.000)	OR: 1.10 (95% CI: 1.07–1.14, p = 0.000)	OR: 1.06 (95% CI: 1.00–1.12, p = 0.052)	−0.05
**Filled a Prescription in the Past 12 Months**	OR: 3.73 (95% CI: 3.46–4.02, p = 0.000)	OR: 3.87 (95% CI: 3.56–4.20, p = 0.000)	OR: 2.50 (95% CI: 2.06–3.03, p = 0.000)	−1.37
**Smoker, Past or Present**	OR: 1.03 (95% CI: 0.97–1.09, p = 0.299)	OR: 1.02 (95% CI: 0.94–1.09, p = 0.685)	OR: 1.04 (95% CI: 0.94–1.15, p = 0.457)	0.02

*Note*. The values presented here are a subset of the larger model. The Unified LR model was developed on 27,626 adult patients including all ages. The Stratified LR: Under 65 was constructed on 19,340 patients, and the Stratified LR: 65+ was based on 8,286 patients. The data for the model were gathered from the 2021 NHIS study.

**Table 4.** Comparison of the Estimated Odds Ratios for Variables that Measure Limitations and Challenges of Ability in the Unified LR, Stratified LR: Under 65, and Stratified LR: 65+ Models

Variable	Unified LR	Stratified LR: Under 65	Stratified LR: 65+	Difference in Odds Ratios
**Communication Difficulty**	OR: 0.92 (95% CI: 0.81–1.05, p = 0.209)	OR: 0.95 (95% CI: 0.79–1.14, p = 0.562)	OR: 0.88 (95% CI: 0.73–1.07, p = 0.214)	−0.06
**Disabled**	OR: 0.94 (95% CI: 0.84–1.05, p = 0.308)	OR: 0.96 (95% CI: 0.81–1.12, p = 0.585)	OR: 0.93 (95% CI: 0.80–1.08, p = 0.347)	−0.03
**Errands Difficulty**	OR: 1.07 (95% CI: 0.95–1.21, p = 0.289)	OR: 1.15 (95% CI: 0.97–1.37, p = 0.106)	OR: 1.00 (95% CI: 0.84–1.19, p = 0.979)	−0.16
**Hands Fingers Difficulty**	OR: 1.09 (95% CI: 0.98–1.21, p = 0.110)	OR: 1.08 (95% CI: 0.92–1.27, p = 0.323)	OR: 1.13 (95% CI: 0.99–1.30, p = 0.067)	0.05
**Hearing Impairment**	OR: 0.91 (95% CI: 0.82–1.02, p = 0.107)	OR: 0.94 (95% CI: 0.80–1.11, p = 0.478)	OR: 0.88 (95% CI: 0.76–1.02, p = 0.093)	−0.06
**Raising Arms Difficulty**	OR: 0.96 (95% CI: 0.84–1.11, p = 0.626)	OR: 1.06 (95% CI: 0.85–1.32, p = 0.610)	OR: 0.91 (95% CI: 0.75–1.10, p = 0.316)	−0.15
**Self-Care Difficulty**	OR: 1.21 (95% CI: 1.03–1.42, p = 0.019)	OR: 1.04 (95% CI: 0.83–1.32, p = 0.712)	OR: 1.33 (95% CI: 1.07–1.66, p = 0.010)	0.29
**Uses Mobility Equipment**	OR: 1.12 (95% CI: 0.99–1.27, p = 0.060)	OR: 1.24 (95% CI: 1.01–1.52, p = 0.038)	OR: 1.11 (95% CI: 0.95–1.29, p = 0.194)	−0.14
**Visual Impairment**	OR: 0.99 (95% CI: 0.92–1.06, p = 0.780)	OR: 1.01 (95% CI: 0.92–1.11, p = 0.868)	OR: 0.96 (95% CI: 0.85–1.08, p = 0.516)	−0.05
**Work Limited by a Health Problem**	OR: 1.10 (95% CI: 1.02–1.20, p = 0.019)	OR: 1.06 (95% CI: 0.95–1.18, p = 0.323)	OR: 1.16 (95% CI: 1.03–1.31, p = 0.015)	0.11

*Note*. The values presented here are a subset of the larger model. The Unified LR model was developed on 27,626 adult patients including all ages. The Stratified LR: Under 65 was constructed on 19,340 patients, and the Stratified LR: 65+ was based on 8,286 patients. The data for the model were gathered from the 2021 NHIS study.

**Table 5.** Comparison of the Estimated Odds Ratios for Variables that Indicate Behavioral Health Conditions and Social Needs in the Unified LR, Stratified LR: Under 65, and Stratified LR: 65+ Models

Variable	Unified LR	Stratified LR: Under 65	Stratified LR: 65+	Difference in Odds Ratios
**Anxiety**	OR: 1.59 (95% CI: 1.46–1.73, p = 0.000)	OR: 1.64 (95% CI: 1.48–1.82, p = 0.000)	OR: 1.41 (95% CI: 1.20–1.65, p = 0.000)	−0.23
**Cost is a Barrier to Medical Care**	OR: 0.98 (95% CI: 0.87–1.11, p = 0.784)	OR: 0.99 (95% CI: 0.86–1.14, p = 0.907)	OR: 0.97 (95% CI: 0.69–1.36, p = 0.868)	−0.02
**Depression**	OR: 1.40 (95% CI: 1.29–1.52, p = 0.000)	OR: 1.44 (95% CI: 1.30–1.59, p = 0.000)	OR: 1.30 (95% CI: 1.13–1.51, p = 0.000)	−0.13
**Food Barrier**	OR: 1.00 (95% CI: 0.88–1.13, p = 0.962)	OR: 1.02 (95% CI: 0.88–1.18, p = 0.798)	OR: 0.95 (95% CI: 0.72–1.26, p = 0.716)	−0.07
**Problems Paying Bills**	OR: 1.14 (95% CI: 1.03–1.26, p = 0.009)	OR: 1.10 (95% CI: 0.98–1.23, p = 0.099)	OR: 1.25 (95% CI: 1.02–1.53, p = 0.028)	0.15
**Income Ratio to Poverty Threshold: 3+**	Reference Group	Reference Group	Reference Group	
**Income Ratio to Poverty Threshold: 2–2.99**	OR: 0.82 (95% CI: 0.76–0.89, p = 0.000)	OR: 0.83 (95% CI: 0.75–0.92, p = 0.000)	OR: 0.82 (95% CI: 0.72–0.94, p = 0.004)	−0.01
**Income Ratio to Poverty Threshold: 1–1.99**	OR: 0.77 (95% CI: 0.70–0.83, p = 0.000)	OR: 0.74 (95% CI: 0.66–0.82, p = 0.000)	OR: 0.81 (95% CI: 0.70–0.93, p = 0.002)	0.07
**Income Ratio to Poverty Threshold: 0.5–0.99**	OR: 0.79 (95% CI: 0.70–0.89, p = 0.000)	OR: 0.75 (95% CI: 0.65–0.87, p = 0.000)	OR: 0.85 (95% CI: 0.69–1.05, p = 0.132)	0.1
**Income Ratio to Poverty Threshold: < 0.5**	OR: 0.73 (95% CI: 0.60–0.88, p = 0.001)	OR: 0.75 (95% CI: 0.60–0.93, p = 0.008)	OR: 0.61 (95% CI: 0.39–0.95, p = 0.028)	−0.14

*Note*. The values presented here are a subset of the larger model. The Unified LR model was developed on 27,626 adult patients including all ages. The Stratified LR: Under 65 was constructed on 19,340 patients, and the Stratified LR: 65+ was based on 8,286 patients. The data for the model were gathered from the 2021 NHIS study.

## Results

We implemented the analyses using R (R Core Team 2022). The raw 2021 NHIS data included information on 29,482 patients. A total of 27,626 patients were identified with complete information in all of the variables utilized in this study, of whom 38.9% had utilized telehealth. There were 19,340 patients under 65 years old, and 8,286 patients were at least 65 years old.

## Comparison of Modeling Strategies

Tables 1–5 show comparisons of the unified and stratified logistic regression models by the category of the variables.

Table 1 shows a comparison of the modeling results for the demographic variables. For variables such as age, region, rural geography, education, and in most categories of race and ethnicity, the stratified and unified models show reasonable consistency in the quantitative estimates and statistical significance of the odds ratios. Male patients showed significantly reduced rates of telehealth utilization relative to patients of other genders in the Unified LR model (OR: 0.80, 95% CI: 0.75–0.84, p < 0.001) and in the Stratified LR: Under 65 model (OR: 0.73, CI: 0.68–0.79, p < 0.001). However, for the Stratified LR: 65+ model, the reduction in telehealth utilization for male patients relative to other patients (OR: 0.96, CI: 0.87–1.06, p = 0.433) is highly attenuated and not statistically significant. Patients of Hispanic ethnicity showed a large disparity in their estimated odds ratios for the Stratified LR: 65+ model (OR: 1.59, CI: 1.30–1.95, p < 0.001) relative to the Stratified LR: Under 65 model (OR: 1.10, CI: 0.99–1.22, p = 0.074) and the Unified LR model (OR: 1.17, CI: 1.07–1.29, p = 0.001).

Table 2 elucidates the modeling results related to health conditions. The unified and stratified models produced similar estimates of the odds ratios for a number of variables, such as arthritis, asthma, cancer, chronic fatigue syndrome, dry mouth, and hypertension. For patients with a myocardial infarction (MI) or stroke, the estimated odds ratios were not statistically significant.

In the Stratified LR: Under 65 model, patients with coronary heart disease (CHD) had a higher and statistically significant rate of telehealth utilization (OR: 1.54, CI: 1.22–1.96, p < 0.001), while this value was smaller and not statistically significant in the Stratified LR: 65+ model (OR: 1.10, CI: 0.94–1.28, p = 0.220). In the Unified LR model, patients with CHD had higher rates of telehealth utilization, which was statistically significant (OR: 1.23, CI: 1.08–1.40, p = 0.002).

For patients with chronic obstructive pulmonary disease (COPD), the Unified LR model showed no sizable or significant association with telehealth utilization (OR: 1.03, CI: 0.91–1.17, p = 0.611). However, the Stratified LR: 65+ model showed significantly higher rates of telehealth utilization for patients with COPD (OR: 1.18, CI: 1.00–1.38, p = 0.049), while the Stratified LR: Under 65 model showed modestly lower rates that were not statistically significant (OR: 0.87, CI: 0.72–1.06, p = 0.162).

Diabetes was sizably and statistically significantly associated with higher rates of telehealth utilization in the Unified LR model (OR: 1.26, CI: 1.15–1.38, p < 0.001) and both stratified models. However, the Stratified LR: Under 65 model showed considerably higher associations (OR: 1.33, CI: 1.17–1.52, p < 0.001) relative to the Stratified LR: 65+ model (OR: 1.16, CI: 1.02–1.32, p = 0.020).

Food allergies showed significant positive associations with telehealth utilization in the Unified LR model (OR: 1.12, CI; 1.03–1.23, p = 0.009) and in the Stratified LR: Under 65 model (OR: 1.21, CI: 1.08–1.34, p < 0.001). However, there was a modest negative association (OR: 0.92, CI: 0.78–1.09, p = 0.348) in the Stratified LR: 65+ model, which was not statistically significant.

High cholesterol showed a modestly higher positive association with telehealth in the Stratified LR: 65+ model (OR: 1.11, CI: 1.01–1.23, p = 0.034), but these associations were smaller and not statistically significant in the Stratified LR: Under 65 model (OR: 1.03, CI: 0.94–1.12, p = 0.549) or in the Unified LR model (OR: 1.05, CI: 0.99–1.12, p = 0.120).

Patients with weak or failing kidneys had large and significantly higher rates of telehealth utilization in the Unified LR model (OR: 1.46, CI: 1.25–1.70, p < 0.001) and in the Stratified LR: 65+ (OR: 1.58, CI: 1.30–1.91, p < 0.001). This association was more moderate in the Stratified LR: Under 65 model (OR: 1.26, CI: 0.98–1.60, p = 0.067), with wider confidence intervals and reduced statistical significance.

For patients with a liver condition, the Unified LR model (OR: 1.22, CI: 0.93–1.60, p = 0.161) and Stratified LR: Under 65 model (OR: 1.36, CI: 0.96–1.91, p = 0.079) and over 65 (OR: 1.05, CI: 0.66–1.67, p = 0.835) led to a range of estimates that differed greatly, albeit with a wide range of uncertainty.

Table 3 includes the modeling estimates for a number of additional health-related variables. Body mass index (BMI) did not show significant associations with telehealth utilization for any category in the models, with the range of associations well within the range of uncertainty.

Hospitalization of the patient within the past 3 months showed sizable and significantly higher rates of telehealth utilization in all cases. The estimate for hospitalization was markedly higher for patients in the Stratified LR: Under 65 model (OR: 1.54, CI: 1.35–1.77, p < 0.001) than for patients in the Stratified LR: 65+ model (OR: 1.32, CI: 1.13–1.54, p = 0.001), while the Unified LR model's estimate was between these values: (OR: 1.44, CI: 1.30–1.60, p < 0.001).

Patients without a usual facility for medical care all had similar lower rates of telehealth utilization in terms of the size and statistical significance of the association. Likewise, the number of emergency department (ED) visits in the past 12 months and the number of urgent care visits in the past 12 months showed similarly modest and statistically significantly higher rates of telehealth utilization across the unified and stratified models. Smoking status did not show sizable or significant associations with telehealth utilization in any of the models.

Patients who filled a prescription in the prior 12 months had much higher rates of telehealth utilization. For the Unified LR model, the estimated odds ratio was 3.73 (OR: 3.73, CI: 3.46–4.02, p < 0.001). For patients in the Stratified LR: 65+ model, this odds ratio was still large (OR: 2.50, CI: 2.06–3.03, p < 0.001), but it was considerably smaller than the estimated association in the Stratified LR: Under 65 model (OR: 3.87, CI: 3.56–4.20, p < 0.001).

Table 4 displays the model's results for variables related to a patient's limitations or challenges of ability. A number of variables, such as communication difficulties, disabled status, difficulty with errands, difficulty raising their arms, difficulty using their hands or fingers, hearing impairments, and visual impairments, did not show sizable associations or variability across the models.

Patients with difficulties related to self-care had a sizable and statistically significantly higher odds of telehealth utilization in the Unified LR model (OR: 1.21, CI: 1.03–1.42, p = 0.019) and in the Stratified LR: 65+ model (OR: 1.33, CI: 1.07–1.66, p = 0.010) but not for the Stratified LR: Under 65 model (OR: 1.04, CI: 0.83–1.32, p = 0.712).

The use of mobility equipment was associated with higher estimated rates of telehealth in the Stratified LR: Under 65 model (OR: 1.24, CI: 1.01–1.52, p = 0.038), while the positive associations in the Stratified LR: 65+ model (OR: 1.11, CI: 0.95–1.29, p = 0.194) and in the Unified LR model (OR: 1.12, CI: 0.99–1.27, p = 0.060) were smaller and with reduced degrees of statistical significance.

Patients whose health problems limited their ability to work had moderately higher rates of telehealth utilization that were statistically significant in the Stratified LR: 65+ model (OR: 1.16, CI: 1.03–1.31, p = 0.015) and in the Unified LR model (OR: 1.10, CI: 1.02–1.20, p = 0.019) but not for the Stratified LR: Under 65 model (OR: 1.06, CI: 0.95–1.18, p = 0.323).

Table 5 includes the modeling results for variables related to behavioral health and social needs. Some factors, such as cost barriers to medical care, food barriers, or poverty ratios in most categories, did not show large gaps between the models.

For patients with anxiety, the Stratified LR: Under 65 model (OR: 1.64, CI: 1.48–1.82, p < 0.001) and Unified LR model (OR: 1.59, CI: 1.46–1.73, p < 0.001) showed somewhat higher estimated odds ratios than the Stratified LR: 65+ model (OR: 1.41, CI: 1.20–1.65, p < 0.001). Likewise, for patients with depression, the estimated odds ratios were somewhat higher in the Stratified LR: Under 65 model (OR: 1.44, CI: 1.30–1.59, p < 0.001) and the Unified LR model (OR: 1.40, CI: 1.29–1.52, p < 0.001) than for the Stratified LR: 65+ model (OR: 1.30, CI: 1.13–1.51, p < 0.001).

For patients with problems paying bills, the estimated rate of telehealth utilization was higher and more statistically significant for Stratified LR: 65+ model (OR: 1.25, CI: 1.02–1.53, p = 0.028) than for the Stratified LR: Under 65 model (OR: 1.10, CI: 0.98–1.23, p = 0.099), while the Unified LR model also demonstrated a moderately large and statistically significant association (OR: 1.14, CI: 1.03–1.26, p = 0.009).

The variables for which the modeling results and conclusions differ based on the method are described in Table 6. This table shows the direction of increase in the estimated odds ratio for telehealth associated with each factor, either towards patients at least 65 years old or toward younger patients. Table 6 also demonstrates when the stratified modeling strategy differs from the unified model in its statistical conclusions. Clear differences arise when the gaps in the odds ratios are large or if the statistical conclusions would change, while smaller gaps in the size of the association or the degree of statistical significance are marked as somewhat different. The Stratified LR: 65+ model shows higher rates of telehealth utilization associated with a number of variables, including male gender, Hispanic ethnicity, COPD, high cholesterol, weak or failing kidneys, difficulty with self-care, health problems that limit the ability to work, and problems paying bills. Meanwhile, the Stratified LR: Under 65 model shows higher estimated odds ratios for variables including CHD, food allergies, liver conditions, patients using mobility equipment, and patients who have filled a prescription within 12 months.

**Table 6.** A Summary of Findings for Variables that Differ Between the Unified LR, Stratified LR: Under 65, and Stratified LR: 65+ Models

Variable	Quantitative Disparity, with Increases in Estimated Odds Ratio Toward	Differences: Unified LR and Stratified LR: Under 65	Differences: Unified LR and Stratified LR: 65+
**Male**	Patients At Least 65	No	Yes
**Hispanic Ethnicity**	Patients At Least 65	Somewhat	Yes
**CHD**	Patients Under 65	Yes	Somewhat
**COPD**	Patients At Least 65	Somewhat	Yes
**Food Allergy**	Patients Under 65	No	Yes
**High Cholesterol**	Patients At Least 65	No	Somewhat
**Kidneys Weak or Failing**	Patients At Least 65	Yes	No
**Liver Condition**	Patients Under 65	Somewhat	Somewhat
**Self-Care Difficulty**	Patients At Least 65	Yes	Somewhat
**Uses Mobility Equipment**	Patients Under 65	Yes	No
**Work Limited by a Health Problem**	Patients At Least 65	Somewhat	No
**Problems Paying Bills**	Patients At Least 65	Somewhat	Somewhat
**Filled a Prescription in the Past 12 Months**	Patients Under 65	No	Yes

## Factors Associated with Telehealth Utilization

The logistic regression models can aid in prediction as well as in inference. The Unified LR model included an in-sample classification accuracy of 69.2% relative to a baseline accuracy of 61.1% (the larger of the two categories) and an area under the receiver-operating characteristic curve (AUC) of 0.749. The Stratified LR: Under 65 model had a classification accuracy of 71.2% relative to a baseline accuracy of 62.6% and an AUC of 0.771. The Stratified LR: 65+ model had a classification accuracy of 65.0% relative to a baseline of 57.7% and an AUC of 0.694.

The models show a range of disparities based on demographic characteristics. The rates of telehealth utilization were significantly lower with older age, and they were significantly higher for patients with higher degrees of education. Relative to other genders, male patients (Unified LR Model: OR: 0.80, CI: 0.75–0.84, p < 0.001) had considerably lower rates of telehealth utilization. Telehealth utilization did not significantly vary by categories of race/ethnicity, with the exception of Hispanic ethnicity. Geographic regions showed significant disparities in telehealth utilization, with patients living in the West having higher rates (Unified LR Model: OR: 1.22, CI: 1.12–1.32, p < 0.001) than the Northeast, while the South (Unified LR Model: OR: 0.78, CI: 0.72–0.84, p < 0.001) and Midwest (Unified LR Model: OR: 0.68, CI: 0.62–0.74, p < 0.001) had sizably lower rates. Patients living in rural areas (Unified LR Model: OR: 0.60, CI: 0.55–0.65, p < 0.001) also had large gaps in utilization.

Many of the health conditions were associated with higher rates of telehealth utilization in the Unified LR model, including arthritis, asthma, CHD, cancer, chronic fatigue syndrome, diabetes, dry mouth, food allergies, and weak or failing kidneys. Selected other health conditions, such as COPD and high cholesterol, showed significant associations only in the Stratified LR: 65+ model. Health utilization, including hospitalization within 3 months, ED visits within 12 months, urgent care visits within 12 months, or filling a prescription medicine within 12 months, were all associated with higher rates of telehealth utilization in the Unified LR model. Meanwhile, lacking a usual facility for medical care was associated with a sizably lower rate of telehealth utilization.

Among the limitations and challenges that patients faced, difficulties with self-care and health problems that limit the ability to work were associated with higher rates of telehealth utilization. These associations were driven by significant results that only appeared in the Stratified LR: 65+ model. Meanwhile, the use of mobility equipment was associated with significantly higher rates of telehealth utilization only in the Stratified LR: Under 65 model.

Behavioral health conditions such as anxiety and depression were each associated with higher rates of telehealth utilization. Among social needs, problems paying bills were associated with moderately higher rates of telehealth utilization, which was driven by a significant effect in the Stratified LR: 65+ model. Incomes below three times the poverty threshold were associated with significantly lower rates of telehealth utilization.

## Discussion

Multivariable statistical modeling is a fundamental method for understanding the trends in telehealth utilization. This study provides a comparison of the unified and age-stratified approaches to modeling telehealth utilization with logistic regression. While a range of factors show similar estimates with either modeling strategy, clear differences arise across a number of variables, especially male gender, Hispanic ethnicity, CHD, COPD, food allergies, high cholesterol, weak or failing kidneys, liver conditions, difficulty with self-care, the use of mobility equipment, health problems that limit the ability to work, problems paying bills, and filling a prescription within the past 12 months. The results of this investigation demonstrate the confounding influence of older age in models of telehealth utilization. Because older patients use and adopt technology at different rates relative to younger patients, it is difficult to accurately estimate the trends in telehealth utilization with simple adjustments for older age. Stratified modeling by age groups allows the models to estimate differing degrees of association for important variables that influence the utilization of telehealth.

Further investigation in this area can improve the quality and precision of statistical models of telehealth utilization. Incorporating the methods of causal inference may better account for the confounding influence of older age and facilitate more accurate estimates of associations in other variables. Other confounding variables may require further stratification of the models. For instance, patients of Hispanic ethnicity showed a large gap in the Stratified LR: 65+ model relative to the Stratified LR: Under 65 model. This disparity may be confounded by additional factors such as geographic region and rural geography.

Many of the health conditions were associated with higher rates of telehealth utilization. This could suggest that telehealth is becoming part of the practice of managing conditions, especially chronic conditions that require regular follow-up. The observed associations between telehealth and categories of health utilization, including urgent care appointments, ED visits, and hospitalization, would also indicate that patients with higher needs are employing telehealth as part of their overall care. Notably, patients who filled at least one prescription in the past 12 months had markedly higher rates of telehealth utilization by a factor of 2.50 (Stratified LR: 65+ model) to 3.87 (Stratified LR: Under 65 model). This could suggest that telehealth may be a key method of helping patients obtain or refill a prescription. Likewise, it may also suggest that patients who do not fill prescriptions have very limited utilization of telehealth.

A variety of social needs and demographic factors were associated with significantly lower rates of telehealth utilization. Patients in poverty, of older age, and those living in rural areas all had reduced rates of telehealth utilization. In many cases, these variables have a compound association, creating groups of patients with few opportunities to utilize telehealth. Prioritizing technological access for all patients should be a priority for the development of more robust and universally available telehealth services.

As a limitation, this study was based upon self-reported data on telehealth utilization and patient characteristics. At the time of this article's preparation, the 2021 data was the most recent currently available from the NHIS. Given the changing circumstances of the COVID-19 pandemic, we expect that trends in the utilization of telehealth may continue to change over time. Based on the large associations of geographical variables and prescription medication usage to telehealth utilization shown in this study, further research in this area could explore the potential of additional stratification in statistical modeling.
